# Genotype combination contributes to psoriasis: An exhaustive algorithm perspective

**DOI:** 10.1371/journal.pone.0186067

**Published:** 2017-10-11

**Authors:** Jinfa Dou, Huimin Guo, Fang Cheng, Hequn Huang, Liying Fu, Longnian Li, Chao Yang, Lei Ye, Leilei Wen, Yuyan Cheng, Lili Tang, Caihong Zhu, Zhengwei Zhu, Wenjun Wang, Yujun Sheng, Zaixing Wang, Shengxiu Liu, Xing Fan, Xianbo Zuo, Fusheng Zhou, Liangdan Sun, Xiaodong Zheng, Xuejun Zhang

**Affiliations:** 1 Institute of Dermatology and Department of Dermatology at No. 1 Hospital, Anhui Medical University, Hefei, China; 2 Key Laboratory of Dermatology, Anhui Medical University, Ministry of Education, Hefei, China; 3 The Department of Dermatology, The First Affiliated Hospital, Gannan Medical University, Ganzhou, China; University of Texas Health Science Center at Houston, UNITED STATES

## Abstract

Researchers have learned that nearly all conditions and diseases have a genetic component. With the benefit of technological advances, many single-nucleotide polymorphisms (SNPs) have been found to be associated with the risk of complex disorders by using genome wide association studies (GWASs). Disease-associated SNPs are sometimes shared by healthy controls and cannot clearly distinguish affected individuals from unaffected ones. The combined effects of multiple independent SNPs contribute to the disease process, but revealing the relationship between genotype and phenotype based on the combinations remains a great challenge. In this study, by considering the disease prevalence rate, we conducted an exhaustive process to identify whether a genotype combination pattern would have a decisive effect on complex disorders. Based on genotype data for 68 reported SNPs in 8,372 psoriasis patients and 8,510 healthy controls, we found that putative causal genotype combination patterns (CGCPs) were only present in psoriasis patients, not in healthy subjects. These results suggested that psoriasis might be contributed by combined genotypes, complementing the traditional modest susceptibility of a single variant in a single gene for a complex disease. This work is the first systematic study to analyze genotype combinations based on the reported susceptibility genes, considering each individual among the cases and controls from the Chinese population, and could potentially advance disease-gene mapping and precision medicine due to the causality relationship between the candidate CGCPs and complex diseases.

## Introduction

It is well known that Mendelian disorders exhibit a recognizable inheritance pattern (classic Mendelian segregation pattern), and the presence of a phenotype is highly correlated with the genotype at the disease causal locus. However, complex diseases are typically considered to be caused by the interaction of multiple genes with each other and with environmental factors [1] in a multifactorial inheritance pattern. Genome-wide association studies (GWASs) and other approaches have been used to investigate the genetic architecture of complex traits and diseases with the production of 21,821 SNP-trait associations recorded in the National Human Genome Research Institute (NHGRI) GWAS Catalog (https://www.ebi.ac.uk/gwas/search?query=*&filter), revealing that genetic factors play an important role in determining susceptibility to complex diseases.

With the development of genotyping and sequencing technologies, perfect genotype information can be obtained for individuals, and susceptibility loci have been identified by comparing the frequency of a single SNP in a case-control comparison, derived from population-based studies [2], including psoriasis cohort studies. Psoriasis is a complex immune-mediated genetic disease typically involving the skin or joints or both, causing physical and psychological burdens on the affected individuals [3]. The etiopathogenesis of psoriasis is complex and still largely unknown. It is generally believed that both genetic and environmental factors contribute to disease susceptibility, and many susceptibility loci/genes have been identified through GWASs [4–10], large-scale meta-analyses of GWASs [11–14] and next-generation sequencing-based association analysis [15]. The clustering of the genetic associations identified to date appears to show involvement in various biological processes, including the IL-23/Th17 pathway, NF-κB signaling, epidermal cell differentiation and so on. All of these susceptibility genes are of potential importance in the pathogenesis of psoriasis through different pathways.

Notably, most of these association studies have focused on a single SNP or one gene, and the findings are obtained from population genetics, ignoring genotype interactions. However, multiple genes may increase the risk and are responsible for the pathogenesis of a disease through their combined effects [16]. The combined genotype approach has high potential utility in association studies [17], and multi-genotype combination analysis is therefore important in studying the pathogenesis and inheritance patterns of psoriasis. To date, however, no systemic genotype combination of multiple susceptibility genes in an exhaustive case-control comparison of individuals has been undertaken. Meanwhile, two major rationales regarding genetic variation lay the foundation for our genotype combination analysis in complex diseases. One is that complex diseases are caused by multiple genes in a specific genotype pattern, and the other is that the sum of the frequencies of all causal genotype patterns in a population will be approximately equal to the prevalence of the disease. In this study, we proposed an exhaustive algorithm to fully screen the genotype combinations of selected genes based on the detection of established psoriasis-associated SNPs in 8,372 psoriasis patients and 8,510 healthy controls, to explore whether there is a specific pattern of polygenic inheritance for complex disorders, such as psoriasis, which is comparable to the classic monogenic inheritance of Mendelian disorders mentioned above, co-segregating with the disease phenotype.

## Materials and methods

### Subjects

The samples included in this study were collected through collaboration with multiple hospitals in China. In total, 8,372 psoriasis patients and 8,510 healthy controls were subjected to genotyping and subsequent analysis. Psoriasis patients were diagnosed by at least two dermatologists, and clinical information was collected through a comprehensive clinical check-up by professional investigators. All healthy controls were clinically assessed to be without psoriasis, any autoimmune disorders and systemic disorders and any family history of psoriasis or other autoimmune-related disorders (including first-, second- and third-degree relatives). Demographic information was collected from all the participants, both cases and controls, through a previously described structured questionnaire [4]. Characteristics of our study samples are summarized in Table 1. The cases and controls were well matched for age, gender and ethnicity. All samples were self-reported Han Chinese. The study was approved by the institutional ethics committee of The First Affiliated Hospital of Anhui Medical University, according to Declaration of Helsinki principle, and all subjects gave informed consent for their participation.

**Table 1:** The summary information of samples.

	Case	Control
No.^a^	8310	7243
Gender		
Male (%)	5008(60.26)	3682(50.84)
Female (%)	3302(39.74)	3561(49.16)
Age		
Mean (s.d.)	35.03(14.75)	31.17 (13.41)
Age of onset		
Mean (s.d.)	27.14(13.37)	-

^a^, the number of subjects with demographic and medical information.

s.d., standard deviation. Mean, the mean value of age/age onset.

### SNPs selection and genotyping

To comprehensively evaluate psoriasis-associated loci based on a combination method, we selected variants that have been reported to be associated with psoriasis based on the following quality criteria: (1) minor allele frequency (MAF) higher than 0.05 in both cases and controls; (2) SNPs with a significant association of P < 5 × 10^−8^ reported in the previous studies; and (3) suitability for genotyping using the Sequenom MassArray system (Sequenom, San Diego, CA) in a batch processing. In total, 68 SNPs in 38 distinct loci listed in S1 Table were selected for genotyping and subjected to a filtering procedure based on the quality of genotyping. The Hardy–Weinberg equilibrium (HWE) in the controls was P > 0.01, and the HWE in the cases was P > 10^−4^. Therefore, quality control on genetic clusters for all the SNPs was carefully considered, and three genotypes of each well-defined SNP were subjected to genotype combination analysis.

### Genotype combination analysis

To determine whether there were causal genotype combination patterns, we performed an exhaustive genotype-combination model search incorporating different numbers of qualified SNPs. Upon exhaustively enumerating each combination of genotypes considered jointly, the total number of genotype combination patterns for the selected SNPs was calculated as follows:

$$\text{Genotype}\;\text{combination}\;\text{patterns}\;=3^{r}\frac{n!}{\left(n-\text{r}\right)!\left(\text{r}\right)!}$$

where r is the number of selected SNPs assigned to produce genotype combinations (for example, ranging from one to n), and n is the total number of SNPs. The invariant constant number three means that each SNP has three genotypes, AA, AB and BB, for the characters A and B denoting two alleles. Note that the total number of genotype combination patterns is all the possibilities of r across n of the the total number of SNPs used in the study. Learning from the method of definition for the causal genotype co-segregated with phenotype in monogenic disease. We assumed that the candidate causal genotype combination patterns (CGCP) played a decisive role in the occurrence of psoriasis, which was present in the cases but not in the controls. In the search process, all the genotype combination patterns would be searched in each individual among the cases and controls. Only candidate CGCPs were picked up, while the others, even if they occurred only once in the controls, would be eliminated from the process.

After eliminating the patterns existing in the control cohort, the remaining patterns would be examined by frequency according to the prevalence rate as a boundary condition to limit the scale of calculation. Given that the coverage of candidate CGCP, if any, would be limited in the complex disease due to its uniqueness, the frequency would be considered to filter the models departing from the prevalence rate of psoriasis. If we assumed the number of cases is m, control is n, the known prevalence rate is p, and x is the magnification times for control, then m/(m+nx) is equal to p. For the obtained genotype data, we defined the frequency of the selected genotype (AA, AB or BB) of SNP_i_ in the case cohort as *α*_*i*_, with β_*i*_ in the control cohort. the realistic frequency of a given genotype F(gi) in the overall population is represented as F(gi) = (m*αi+nxβi)/(m+nx). When we set λ = (1-p)/p, the formula is inductively defined as follows:

$$\text{F}\left(\text{gi}\right)\;=\;\left(\alpha_{i}+\lambda \beta_{i}\right)/\left(1+\lambda \right)$$

where λ is a constant related to the prevalence of psoriasis for 4.7‰ [18]. Based on the data from our genotyping group, the frequency of any CGCP was less than the prevalence of 4.7‰ as a preliminary filter. It is supposed that there are 5 to 100 CGCPs in an affected subject for a complex disease, which signifies a 1/100 to 1/5 prevalence for the contribution of one CGCP, so to strictly control boundary conditions with practical significance, we refined the frequency of CGCP as shown by the following formula:

$$0.01\;\text{P}<\prod_{i=1}^{r}\text{F}(\text{gi})<0.2\;\text{P}$$

where P is the prevalence rate of psoriasis, and we set P = 4.7‰ as mentioned above in this study.

### Permutation test

Permutation test was used to shuffle the phenotypes randomly to generate distributions of test samples with a specific genotype combination. We performed 1000 permutations for 1000 cases randomly sampled from the observed patients to the top candidate CGCP and sequentially counted the samples with candidate CGCP to make the frequency distribution.

### Inverse genotype combination analysis

When we turn to consider cases and controls, an obvious question is whether genotype combination patterns specifically determine the healthy phenotype. Consequently, we postulate a null hypothesis that healthy individuals are “patients” with respect to “psoriasis controls” with a prevalence of 99.53%. An inverse genotype combination analysis was performed through exhaustively searching the whole cohort, and the combination patterns existing only in the “patient cohort” were retained. We set the boundary conditions using the same criteria mentioned above. Because the prevalence of putative “patients” is close to 1, we confined the frequency of genotype combination between 0.01 and 0.2.

## Results

### Genotyping

**Fig 1:**
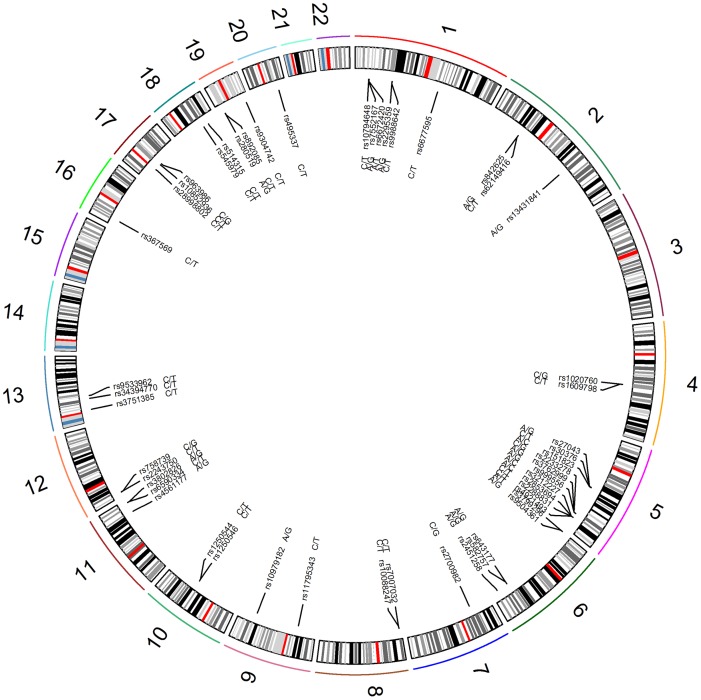
The 51 SNPs and alleles associated with psoriasis in the autosomal chromosome. The outmost lines with different colors represent chromosomes labled by 1–22, and ribbon colors in chromosomes assigned to Giemsa stain based on the color scheme of the UCSC genome browser represent chromosome bands.

Reviewing the genotype data of all the SNPs, 51 passed quality control filtering (Fig 1) and the genotyping information for the studied individuals were provided in S2 Table. Based on the individual genotype of the study cohort consisting of 8,372 psoriasis patients and 8,510 healthy controls for the eligible SNPs, we performed genotype combination analysis using affected and unaffected individuals to identify the candidate CGCPs by fully screening every pattern exhaustively.

### CGCP survey

When we used our exhaustive algorithm to perform genotype combination analysis for three SNPs, more than 120 candidate CGCPs were generated for which at least eight patients specifically harboring one of them with empirical significance. A normal distribution was obtained according to the frequency of all the candidate CGCPs. Then, we added SNPs one by one to investigate the candidate CGCPs for four and five SNPs and obtained an increased number, which suggested that more variants had much more potential to distinguish patients from healthy controls in the early stage. Notably, the average frequency of all candidate CGCPs with empirical significance declined step by step (S3–S5 Tables), which suggested that the candidate CGCPs associated with psoriasis would have a boundary of total SNP number.

**Fig 2:**
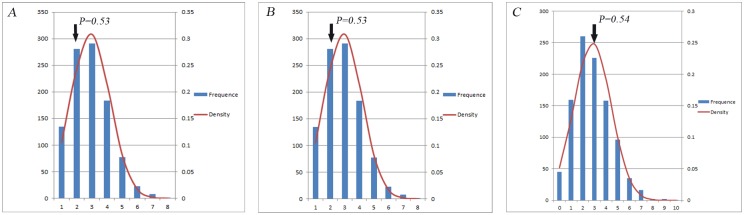
Distribution of permuted number of patients with the candidate CGCP in every 1000 cases. The arrow shows the observed number of individuals with the toppest candidate CGCP for three (A), four (B) and five (C) SNPs in the 1000 cases.

When we turned our attention to the highest capacity of patients for the candidate CGCPs, we found a different number of specific affected individuals. For three SNPs, we obtained the maximum of 16 out of 8,372 patients with the genotype combination of SNPs rs10794648, rs2233278 and rs280519, referring to CCGGGG. The genotype combination of the four SNPs rs367569, rs3751385, rs758739 and rs999556, CCTTGGAA, called the maximum number of affected individuals at 19. Likewise, the CGCPs for TCGCAATCAG, derived from the five SNPs rs10852936, rs2233278, rs4561177, rs6590334 and rs7552167, accounted for the 24 harboring individuals suffering from psoriasis. The toppest candidated CGCP results are shown in Table 2 for each selected number of SNPs. In order to test the number of patients with these CGCPs is beyond what might be expected by chance, we did permutation test at 1000 times to observe the distribution of the number of patients in every 1000 permuted cases. As Fig 2 shows, the observed CGCP individuals are robust in cases.

**Table 2:** The toppest candidate CGCP results for three, four and five SNPs and corresponding number of psoriasis patients.

SNPs					Candidate CGCPs	No. of patients
**3 SNPs**						
rs10794648	rs2233278	rs280519			CCGGGG	16
**4 SNPs**						
rs367569	rs3751385	rs758739	rs999556		CCTTGGAA	19
**5 SNPs**						
rs10852936	rs2233278	rs4561177	rs6590334	rs7552167	TCGCAATCAG	24

Abbreviations: SNP, single nucleotide polymorphism; CGCP, causal genotype combination pattern.

### Reverse CGCP survey

**Fig 3:**
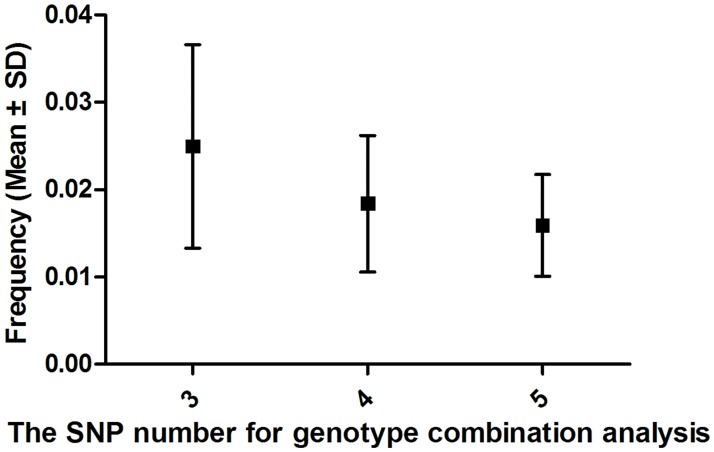
Frequency of genotype combination patterns for three, four and five SNPs in inverse analysis.

To test the robustness of our computational models for exploring the causal effect of specific genotype combinations, we subsequently moved our focus to healthy controls and conducted an inverse genotype combination analysis. The results were notably comparable to the CGCP analysis. When the number of SNPs for inverse genotype combination analysis was 3, 4 and 5, we found plenty of “healthy patients-causal genotype combination patterns” covering 44, 52 and 57 “healthy patients” respectively, at most (“healthy patients” is a special term relative to “psoriasis controls”). As expected, the frequency of these genotype combination patterns decreased as the SNPs included in the search model increase (Fig 3). When the SNP number increased to a certain definite value, the result of “healthy patients-causal genotype combination patterns” would be beyond the minimum of filter conditions. In other words, the findings would be obtained with limited genetic models for “healthy patients” compared to “psoriasis controls”. On the contrary, the CGCPs in psoriasis would reach the boundary with more SNPs included. This result means that the relative control, to a certain case, is less complex than the complicated disease, which is contrary to the actual healthy control situation in genetic association studies (individuals with other diseases or health conditions could be considered as controls relative to psoriasis). Therefore, our hypothesis that healthy individuals are “patients” has been rejected, and these results suggested that so-called healthy control individuals were “relative controls” with regard to a specific disease.

## Discussion

Studies have shown that complex interactions between genes and environmental factors might account for disease risk through altering the function and expression of related genes [19, 20], implicating genetic variations as susceptibility factors for the penetrance, age of onset, severity, and progression of a disease triggered by environmental exposure. However, the causal individual-attributable risk remains indefinable due to the majority of variations co-existing between cases and controls with different frequencies based on single-gene polymorphism association studies. In this study, we representatively selected 68 SNPs from 38 loci or genes to genotype using the Sequenom MassArray, then performed a CGCP analysis for the 52 qualified SNPs through a local exhaustive approach, considering extreme genotype combinations with decisive effects on psoriasis. To our knowledge, this study is the first exhaustive search for the candidate CGCPs in affected individuals, filtering non-CGCPs existing in healthy controls, to obtain the contributors to psoriasis in a genotype combination manner and further to precisely predict whether the disease will arise or not in the future in the subpopulations possessing one of the CGCPs.

For complex traits, there has been no specific correlation between genotype and the clinical phenotype. Few studies have investigated the joint effect of diverse SNPs in individuals, other than a few genotype combination association studies [21, 22] and prediction studies based on several known related SNPs [16, 23], without fully and exhaustively searching for the genotypes of susceptibility SNPs. It is unknown beforehand whether a specific genotype combination exists in psoriasis patients to exert a decisive effect as a major contributor; hence, our proposal to apply genotype combination analysis with an exhaustive algorithm is a robust choice which could contain all the possible scenarios to survey CGCPs. When we utilized three, four and five SNPs to construct genotype combinations, to our surprise, we obtained a wide variety of candidate CGCPs with potential biological significance that would confer an absolute risk effect on their carriers, which to some extent interpreted the high prevalence of psoriasis [18]. However, the number of SNPs in the CGCP would have an upper limit based on our analysis due to the prevalence of psoriasis, like the bounded gaps between primes [24]. Simultaneously, when converting psoriasis patients and controls to perform inverse genotype combination analysis, we found that the genotype combination of healthy controls fitted our model well, and the control samples were relatively unaffected individuals.

**Fig 4:**
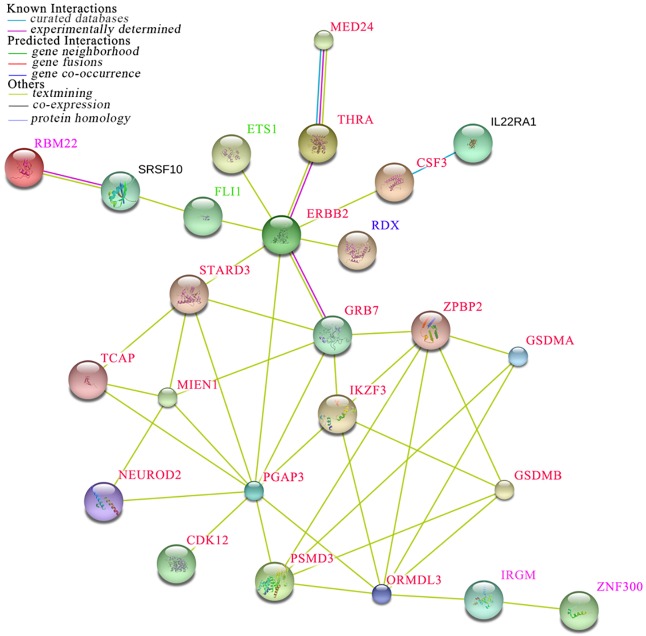
Protein–protein interaction network of the genes at the loci of the 5 SNPs for the top CGCP. Text in red, pink, blue, green and black represents genes at the loci of the 5 SNPs rs10852936, rs2233278, rs4561177, rs6590334 and rs7552167, respectively.

Notably, most candidate CGCPs were derived from the homozygous genotypes of selected SNPs, and the homozygotes of specific SNPs were more likely to have a determinate risk of psoriasis through homozygous mutation of each SNP as well as combinations with each other. The frequency of each candidate CGCP in the overall population was low, less than 0.3%, suggesting that rare combinations could play a causal role in disease initiation or progress, which was consistent with the observation that the mutation rate of causal gene would occur at very low frequency in a monogenic disease, such as the *MVK* mutation rate of 1% for the single causal nucleotide mutation in individuals with sporadic disseminated superficial actinic porokeratosis, an autosomal dominantly inherited disease [25]. Our genotype combination analysis using an exhaustive algorithm differed from traditional linkage and association studies, and the results provided reliable evidence that the combined effects of genetic variants of the susceptibility or candidate causal genes were a determining factor for a particular cohort of psoriasis. No genotype combination analysis of complex diseases has been previously reported in the literature, and therefore the exact molecular mechanism of the jointly decisive role remains elusive. However, given their potential link to psoriasis, it is possible that the candidate CGCPs synthetically change the expression of corresponding genes or have an interaction effect in the pathogenesis pathway through mutations in specific genes. These results suggested the importance of genotype combinations in complex diseases, presumably because one gene’s function relies on the context of other genes [26]. When we mapped the genes in the 400 kb region of the 5 SNPs for the top candidate CGCP to STRING database to acquire protein-protein interactions [27], we found they constructed an interaction network, which suggested that these implicated genes together play an important role in the pathogenesis of psoriasis (Fig 4).

We have revealed the special influence pattern of psoriasis, and the carriers of these candidate CGCPs suffer from psoriasis, complementing the traditional explanation of a multifactorial inheritance pattern of complex disease involving the interaction of multiple genes and environmental factors. We refined the results through enumerative algorithms followed by comprehensive comparison and the consideration of biological significance. In fact, we have proposed a new strategy to study complex diseases by combining genotype information with phenotype information and found there were subpopulations within a disease cohort, as a special group, probably influenced by causal genotype combinations. It is well known that the variants underlying Mendelian diseases are generally highly penetrant, and the affected patient typically has a certain genotype, almost beyond influence by the environment. Furthermore, the boundary of rare Mendelian and common complex diseases has been broken down as the comorbidity associations between them have been revealed [28]. Our study is a result of genetic lessons learned from Mendelian disease and extends our knowledge of the multifactorial inheritance pattern of psoriasis. Systemic CGCP analysis will therefore become increasingly important for understanding the pathogenesis underlying complex diseases.

One of the limitations of our study is the small sample size and limited number of reported SNPs. Moreover, not all the candidate CGCPs were evaluated due to the complexity of computation. Thus, an additional replication study is needed in a much larger sample to validate our results. Furthermore, because the genetic structure of the identified alleles may vary in different ethnic populations, additional studies are needed to test the applicability of our findings to other ethnic populations. It is necessary to note that there might be false genotyping data due to technical limitations, such as an equivocal fluorescent signal for some genotype. Therefore, much more accurate genotype data are needed to perform this analysis for precise medicine research in the future.

In conclusion, we systematically conducted a genotype combination analysis based on the reported psoriasis-associated SNPs using an exhaustive search, providing a new perspective for the basis of complex diseases. A better understanding of disease susceptibility genes in conjunction with genotype combinations will undoubtedly promote our understanding of the pattern of inheritance of complex diseases with a genetic basis and further facilitate studies on pathogenesis, which undoubtedly has a high potential for contributing to precision medicine for psoriasis. However, understanding how causal genotype combinations promote the development of psoriasis will require comprehensive and systematic follow-up of genetic association and functional studies.

## Supporting information

S1 Table Selected SNPs associated with psoriasis were subjected to genotyping. (DOCX)

S2 Table The genotyping information for the qualified SNPs in the studied population. (XLSX)

S3 Table The candidate CGCP results derived from three SNPs, denoted as SNP combination followed by genotype combination, the number of corresponding psoriasis patients and the frequency in the general population. (XLSX)

S4 Table The candidate CGCP results derived from four SNPs, denoted as SNP combination followed by genotype combination, the number of corresponding psoriasis patients and the frequency in the general population. (XLSX)

S5 Table The candidate CGCP results derived from five SNPs, denoted as SNP combination followed by genotype combination, the number of corresponding psoriasis patients and the frequency in the general population. (XLSX)
