# Supplementary material for: Genotype combination contributes to psoriasis: An exhaustive algorithm perspective
Source: PLoS One. 2017 Oct 11;12(10):e0186067. doi: 10.1371/journal.pone.0186067 (PMC5636117; doi:10.1371/journal.pone.0186067)
Supplement: S1 Table — (DOCX) [file pone.0186067.s001.docx]

S1 Table. The selected SNPs associated with psoriasis were subjected to genotype.

| SNP | Chr. | Position (bp) | Notable gene | Minor allele | Function | F_A | F_U | F_O | Ref. |
| --- | --- | --- | --- | --- | --- | --- | --- | --- | --- |
| rs9988642 | 1 | 67726104 | *IL23R* | C | downstream | 0.00024 | 0.000414 | 0 | ^[^[^1^](#_ENREF_1)^]^ |
| rs12564022 | 1 | 67670755 | *IL23R* | C | intronic | NA | NA | 0.4907 | ^[^[^2^](#_ENREF_2)^]^ |
| rs2295359 | 1 | 67635950 | *IL23R* | A | intronic | 0.359 | 0.3458 | 0.4029 | ^[^[^2^](#_ENREF_2)^]^ |
| rs10794648 | 1 | 24518206 | *IL28RA* | T | intergenic | 0.3004 | 0.3314 | 0.3835 | ^[^[^2^](#_ENREF_2)^]^ |
| rs7552167 | 1 | 24518643 | *IL28RA* | A | intergenic | 0.2063 | 0.239 | 0.2718 | ^[^[^1^](#_ENREF_1)^]^ |
| rs7536201 | 1 | 25293084 | *RUNX3* | T | intergenic | 0.2592 | 0.2843 | 0.3058 | ^[^[^1^](#_ENREF_1)^]^ |
| rs6672420 | 1 | 25291010 | *RUNX3* | A | exonic | 0.2571 | 0.2794 | 0.3058 | ^[^[^2^](#_ENREF_2)^]^ |
| rs11121129 | 1 | 8268095 | *SLC45A1,TNFRSF9* | A | upstream | 0.2791 | 0.2831 | 0 | ^[^[^1^](#_ENREF_1)^]^ |
| rs417065 | 1 | 8264112 | *SLC45A1, NFRSF9* | A | intergenic | NA | NA | 0 | ^[^[^2^](#_ENREF_2)^]^ |
| rs6677595 | 1 | 152590187 | *LCE3B, LCE3A* | C | intergenic | 0.3571 | 0.4262 | 0.3786 | ^[^[^1^](#_ENREF_1)^]^ |
| rs4845459 | 1 | 152603842 | *LCE* | C | intergenic | NA | NA | 0.3786 | ^[^[^2^](#_ENREF_2)^]^ |
| rs842625 | 2 | 61080482 | *REL* | A | ncRNA_intronic | 0.1377 | 0.142 | 0.1748 | ^[^[^2^](#_ENREF_2)^]^ |
| rs62149416 | 2 | 61083506 | *FLJ16341, REL* | C | ncRNA_intronic | 0.01861 | 0.01713 | 0.0194 | ^[^[^1^](#_ENREF_1)^]^ |
| rs13431841 | 2 | 163137683 | *IFIH1* | A | intronic | 0.1058 | 0.1239 | 0.1311 | ^[^[^3^](#_ENREF_3)^]^ |
| rs1990760 | 2 | 163124051 | *IFIH1* | T | missense | 0.1949 | 0.1943 | 0.1456 | ^[^[^2^](#_ENREF_2)^]^ |
| rs28512356 | 3 | 189615475 | *TP63* | A | downstream | 0.1812 | 0.1943 | 0.2379 | ^[^[^2^](#_ENREF_2)^]^ |
| rs1020760 | 4 | 103514445 | *NFKB1* | G | intronic | 0.4606 | 0.4396 | 0.4417 | ^[^[^3^](#_ENREF_3)^]^ |
| rs1609798 | 4 | 103537442 | *NFKB1* | T | intronic | 0.3685 | 0.3539 | 0.3398 | ^[^[^3^](#_ENREF_3)^]^ |
| rs151823 | 5 | 96159992 | *ERAP1* | A | intergenic | 0.4798 | 0.5028 | 0.4709 | ^[^[^4^](#_ENREF_4)^]^ |
| rs27043 | 5 | 96117300 | *ERAP1* | G | intronic | 0.4725 | 0.4991 | 0.4757 | ^[^[^3^](#_ENREF_3)^]^ |
| rs30376 | 5 | 96120259 | *ERAP1* | C | intronic | 0.4711 | 0.4971 | 0.4757 | ^[^[^2^](#_ENREF_2)^]^ |
| rs2910686 | 5 | 96252589 | *ERAP2* | C | intronic | 0.3659 | 0.3873 | 0.3738 | ^[^[^2^](#_ENREF_2)^]^ |
| rs1295685 | 5 | 131996445 | *IL13* | A | UTR3 | 0.2805 | 0.3011 | 0.3204 | ^[^[^1^](#_ENREF_1)^]^ |
| rs3762999 | 5 | 150469426 | *TNIP1* | C | intergenic | 0.265 | 0.2254 | 0.1845 | ^[^[^4^](#_ENREF_4)^]^ |
| rs999556 | 5 | 150473674 | *TNIP1* | A | intergenic | 0.2755 | 0.2309 | 0.1893 | ^[^[^4^](#_ENREF_4)^]^ |
| rs2233278 | 5 | 150467189 | *TNIP1* | C | UTR5 | 0.1051 | 0.07437 | 0.0485 | ^[^[^1^](#_ENREF_1)^]^ |
| rs3212227 | 5 | 158742950 | *IL12B* | G | UTR3 | 0.3845 | 0.4322 | 0.432 | ^[^[^5^](#_ENREF_5)^,^ [^6^](#_ENREF_6)^]^ |
| rs2288831 | 5 | 158750013 | *IL12B* | C | intronic | 0.3924 | 0.4387 | 0.4369 | ^[^[^3^](#_ENREF_3)^]^ |
| rs7709212 | 5 | 158764177 | *IL12B* | C | ncRNA_intronic | 0.401 | 0.4432 | 0.432 | ^[^[^2^](#_ENREF_2)^,^ [^7^](#_ENREF_7)^]^ |
| rs4921493 | 5 | 158836107 | *IL12B* | C | intergenic | 0.3121 | 0.3416 | 0.3301 | ^[^[^2^](#_ENREF_2)^]^ |
| rs2853694 | 5 | 158749088 | *IL12B* | G | intronic | 0.3595 | 0.3283 | 0.3252 | ^[^[^2^](#_ENREF_2)^]^ |
| rs9504361 | 6 | 577820 | *EXOC2* | G | intronic | 0.2189 | 0.2411 | 0.2233 | ^[^[^1^](#_ENREF_1)^]^ |
| rs3799296 | 6 | 542416 | *EXOC2, IRF4* | A | intronic | 0.3233 | 0.3536 | 0.3447 | ^[^[^2^](#_ENREF_2)^]^ |
| rs13210247 | 6 | 111922720 | *TRAF3IP2* | G | ncRNA_exonic | 0.05529 | 0.05624 | 0.068 | ^[^[^8^](#_ENREF_8)^]^ |
| rs582757 | 6 | 138197824 | *TNFAIP3* | C | intronic | 0.08093 | 0.07346 | 0.1019 | ^[^[^1^](#_ENREF_1)^]^ |
| rs643177 | 6 | 138195693 | *TNFAIP3* | C | intronic | 0.08012 | 0.07127 | 0.1019 | ^[^[^2^](#_ENREF_2)^]^ |
| rs2451258 | 6 | 159506600 | *TAGAP* | C | intergenic | 0.07389 | 0.07091 | 0.0777 | ^[^[^1^](#_ENREF_1)^]^ |
| rs2700982 | 7 | 37394820 | *ELMO1* | G | intronic | 0.2211 | 0.2326 | 0.233 | ^[^[^2^](#_ENREF_2)^]^ |
| rs7007032 | 8 | 3679446 | *CSMD1* | C | intronic | 0.1991 | 0.1824 | 0.2136 | ^[^[^4^](#_ENREF_4)^]^ |
| rs10088247 | 8 | 3684199 | *CSMD1* | C | intronic | 0.1981 | 0.1823 | 0.2282 | ^[^[^4^](#_ENREF_4)^]^ |
| rs11795343 | 9 | 32523737 | *DDX58* | C | intronic | 0.224 | 0.2357 | 0.2621 | ^[^[^1^](#_ENREF_1)^]^ |
| rs10979182 | 9 | 110817020 | *RPL31P43* | A | intergenic | 0.4842 | 0.4701 | 0.4563 | ^[^[^1^](#_ENREF_1)^]^ |
| rs1250544 | 10 | 81032885 | *ZMIZ1* | A | intronic | 0.4848 | 0.4977 | 0.4854 | ^[^[^9^](#_ENREF_9)^]^ |
| rs1250546 | 10 | 81032532 | *ZMIZ1* | G | intronic | 0.4918 | 0.5046 | 0.4951 | ^[^[^1^](#_ENREF_1)^,^ [^2^](#_ENREF_2)^]^ |
| rs7107446 | 11 | 64103634 | *PRDX5* | G | intergenic | NA | NA | 0.4417 | ^[^[^2^](#_ENREF_2)^]^ |
| rs4561177 | 11 | 109962432 | *ZC3H12C* | A | intergenic | 0.3896 | 0.3782 | 0.3932 | ^[^[^1^](#_ENREF_1)^]^ |
| rs3802826 | 11 | 128406438 | *ETS1* | A | intronic | 0.2869 | 0.2844 | 0.3301 | ^[^[^1^](#_ENREF_1)^]^ |
| rs6590334 | 11 | 128403208 | *ETS1* | T | intronic | 0.199 | 0.1916 | 0.2039 | ^[^[^2^](#_ENREF_2)^]^ |
| rs758739 | 12 | 6626369 | *NCAPD2* | G | intronic | 0.2632 | 0.2851 | 0.267 | ^[^[^3^](#_ENREF_3)^]^ |
| rs2243750 | 12 | 6567787 | *TAPBPL* | C | intronic | 0.3052 | 0.3247 | 0.2961 | ^[^[^3^](#_ENREF_3)^]^ |
| rs3751385 | 13 | 20762956 | *GJB2* | A | UTR3 | 0.5142 | 0.4761 | 0.4612 | ^[^[^4^](#_ENREF_4)^]^ |
| rs34394770 | 13 | 40333369 | *COG6* | C | intronic | 0.2707 | 0.2729 | 0.2573 | ^[^[^2^](#_ENREF_2)^]^ |
| rs9533962 | 13 | 45334194 | *LOC144817* | T | NA | 0.4907 | 0.4752 | 0.4903 | ^[^[^2^](#_ENREF_2)^]^ |
| rs13708 | 16 | 31000809 | *FBXL19* | G | UTR3 | NA | NA | 0.0291 | ^[^[^2^](#_ENREF_2)^]^ |
| rs367569 | 16 | 11365500 | *PRM3,SOCS1* | T | intergenic | 0.123 | 0.1242 | 0.1845 | ^[^[^1^](#_ENREF_1)^]^ |
| rs10852936 | 17 | 38031714 | *IKZF3* | T | intronic | 0.3008 | 0.2709 | 0.3592 | ^[^[^3^](#_ENREF_3)^]^ |
| rs12936231 | 17 | 38029120 | *ZPBP2* | G | intronic | NA | NA | 0.3641 | ^[^[^3^](#_ENREF_3)^]^ |
| rs28998802 | 17 | 26124908 | *NOS2* | A | intronic | 0.02037 | 0.0172 | 0.0097 | ^[^[^1^](#_ENREF_1)^]^ |
| rs963986 | 17 | 40561579 | *PTRF* | C | intronic | 0.319 | 0.3147 | 0.267 | ^[^[^1^](#_ENREF_1)^]^ |
| rs545979 | 18 | 51819750 | *POLI* | T | intronic | 0.0365 | 0.03025 | 0.0291 | ^[^[^1^](#_ENREF_1)^]^ |
| rs514315 | 18 | 61656785 | *SERPINB8* | C | downstream | 0.2441 | 0.2472 | 0.267 | ^[^[^4^](#_ENREF_4)^]^ |
| rs280519 | 19 | 10472933 | *TYK2* | A | intronic | NA | NA | 0.4272 | ^[^[^10^](#_ENREF_10)^]^ |
| rs892085 | 19 | 10818092 | *QTRT1* | A | intronic | 0.3081 | 0.3051 | 0.2816 | ^[^[^1^](#_ENREF_1)^]^ |
| rs9304742 | 19 | 53451291 | *ZNF816-ZNF321P* | C | intronic | 0.3176 | 0.3465 | 0.301 | ^[^[^4^](#_ENREF_4)^]^ |
| rs495337 | 20 | 48522330 | *SPATA2* | A | exonic | 0.3446 | 0.3629 | 0.3495 | ^[^[^11^](#_ENREF_11)^,^ [^12^](#_ENREF_12)^]^ |
| rs1056198 | 20 | 48556229 | *RNF114* | T | intronic | 0.3434 | 0.3602 | 0.3544 | ^[^[^1^](#_ENREF_1)^]^ |
| rs7352944 | 20 | 48632610 | *RNF114* | C | intergenic | NA | NA | 0.4903 | ^[^[^2^](#_ENREF_2)^]^ |
| rs8128234 | 21 | 36470865 | *RUNX1* | T | intergenic | NA | NA | 0.1408 | ^[^[^2^](#_ENREF_2)^]^ |

Note: the position of SNPs based on the *hg19*; bp, base pair; Chr, chromosome; F_A, MAF in cases; F_U, MAF in controls; F_O, MAF in overall population for Han Chinese in Beijing from the 1000 genomes project (phase 3); NA, not available.

**Reference**

1. Tsoi LC, Spain SL, Knight J, et al. Identification of 15 new psoriasis susceptibility loci highlights the role of innate immunity. Nature genetics. 2012;44(12):1341-1348.

2. Yin X, Low HQ, Wang L, et al. Genome-wide meta-analysis identifies multiple novel associations and ethnic heterogeneity of psoriasis susceptibility. 2015;6:6916.

3. Sheng Y, Jin X, Xu J, et al. Sequencing-based approach identified three new susceptibility loci for psoriasis. Nature communications. 2014;5:4331.

4. Sun LD, Cheng H, Wang ZX, et al. Association analyses identify six new psoriasis susceptibility loci in the Chinese population. Nature genetics. 2010;42(11):1005-1009.

5. Cargill M, Schrodi SJ, Chang M, et al. A large-scale genetic association study confirms IL12B and leads to the identification of IL23R as psoriasis-risk genes. American journal of human genetics. 2007;80(2):273-290.

6. Zhu KJ, Zhu CY, Shi G, et al. Meta-analysis of IL12B polymorphisms (rs3212227, rs6887695) with psoriasis and psoriatic arthritis. Rheumatology international. 2013;33(7):1785-1790.

7. Zhang XJ, Huang W, Yang S, et al. Psoriasis genome-wide association study identifies susceptibility variants within LCE gene cluster at 1q21. Nature genetics. 2009;41(2):205-210.

8. Ellinghaus E, Ellinghaus D, Stuart PE, et al. Genome-wide association study identifies a psoriasis susceptibility locus at TRAF3IP2. Nature genetics. 2010;42(11):991-995.

9. Ellinghaus D, Ellinghaus E, Nair RP, et al. Combined analysis of genome-wide association studies for Crohn disease and psoriasis identifies seven shared susceptibility loci. American journal of human genetics. 2012;90(4):636-647.

10. Strange A, Capon F, Spencer CC, et al. A genome-wide association study identifies new psoriasis susceptibility loci and an interaction between HLA-C and ERAP1. Nature genetics. 2010;42(11):985-990.

11. Capon F, Bijlmakers MJ, Wolf N, et al. Identification of ZNF313/RNF114 as a novel psoriasis susceptibility gene. Human molecular genetics. 2008;17(13):1938-1945.

12. Stuart PE, Nair RP, Ellinghaus E, et al. Genome-wide association analysis identifies three psoriasis susceptibility loci. Nature genetics. 2010;42(11):1000-1004.
